# Fibrodysplasia ossificans progressiva: current concepts from bench to bedside

**DOI:** 10.1242/dmm.046441

**Published:** 2020-09-21

**Authors:** Arun-Kumar Kaliya-Perumal, Tom J. Carney, Philip W. Ingham

**Affiliations:** 1 Lee Kong Chian School of Medicine, Nanyang Technological University Singapore, 59 Nanyang Drive, 636921, Singapore; 2 Institute of Molecular and Cell Biology (IMCB), Agency for Science, Technology and Research (A*STAR), 61 Biopolis Drive, Proteos 138673, Singapore

**Keywords:** Bone morphogenetic protein, ACVR1, Fibrodysplasia ossificans progressiva, Heterotopic ossification, Inflammation

## Abstract

Heterotopic ossification (HO) is a disorder characterised by the formation of ectopic bone in soft tissue. Acquired HO typically occurs in response to trauma and is relatively common, yet its aetiology remains poorly understood. Genetic forms, by contrast, are very rare, but provide insights into the mechanisms of HO pathobiology. Fibrodysplasia ossificans progressiva (FOP) is the most debilitating form of HO. All patients reported to date carry heterozygous gain-of-function mutations in the gene encoding activin A receptor type I (ACVR1). These mutations cause dysregulated bone morphogenetic protein (BMP) signalling, leading to HO at extraskeletal sites including, but not limited to, muscles, ligaments, tendons and fascia. Ever since the identification of the causative gene, developing a cure for FOP has been a focus of investigation, and studies have decoded the pathophysiology at the molecular and cellular levels, and explored novel management strategies. Based on the established role of BMP signalling throughout HO in FOP, therapeutic modalities that target multiple levels of the signalling cascade have been designed, and some drugs have entered clinical trials, holding out hope of a cure. A potential role of other signalling pathways that could influence the dysregulated BMP signalling and present alternative therapeutic targets remains a matter of debate. Here, we review the recent FOP literature, including pathophysiology, clinical aspects, animal models and current management strategies. We also consider how this research can inform our understanding of other types of HO and highlight some of the remaining knowledge gaps.

## Abstract (precis)

**Summary:** Fibrodysplasia ossificans progressiva is a rare disease characterised by progressive heterotopic bone formation. Here, we present a comprehensive summary of the recent literature on this debilitating condition and discuss approaches to solving this clinical puzzle.

## Introduction

Skeletal bone formation, also known as osteogenesis or ossification (see Glossary of clinical terms, Box 1), is a multi-step process involving the formation of mature mineralised bone through the differentiation of progenitor cells (Gilbert, 2000). There are two types of ossification, namely intramembranous and endochondral (Box 1), both of which are temporally and spatially regulated (Setiawati and Rahardjo, 2019). Any disruption to this regulation can lead to abnormal skeletal development or heterotopic (extraskeletal) ossification (HO; Box 1).

**Box 1. Glossary of clinical terms**

**Ankylosis:** fusion of a joint, resulting in a complete restriction of its movement.

**Endochondral ossification:** mesenchymal cells form cartilage that proliferates and matures to be replaced eventually by osteoblasts leading to bone formation.

**Fibrodysplasia ossificans progressiva (FOP):** an extremely rare genetic disorder characterised by heterotopic endochondral ossification at multiple sites, predominantly muscles, tendons, ligaments and fascia.

**Flare-up:** onset of heterotopic ossification in FOP in which the underlying acute inflammation causes the appearance of painful and warm soft tissue swellings.

**Hallux valgus:** outward deviation of the great toe.

**Heterotopic ossification (HO):** bone formation at extraskeletal or ectopic sites.

**Intramembranous ossification:** mesenchymal cells directly differentiate into osteoblasts that eventually form bone.

**Macrodactyly:** abnormally large fingers or toes.

**Metamorphosis:** transformation of one tissue into another.

**Osteochondromas:** benign bony protuberances with a cartilaginous cap, mostly originating from the growth plate of long bones.

**Osteogenesis/ossification:** the process of bone formation.

**Thoracic insufficiency syndrome:** inability of the thorax to carry out normal breathing functions.

HO is the formation of bone, either solitary or multiple, in extraskeletal soft tissues of the body. Several genetic diseases manifest with HO at multiple sites, including fibrodysplasia ossificans progressiva (FOP; Box 1), progressive osseous heteroplasia (POH) and Albright's hereditary osteodystrophy (AHO) (Shore and Kaplan, 2010). Among these, FOP is characterised by HO of endochondral origin, predominantly at muscles, tendons, ligaments and fascia (Kaplan et al., 2008), whereas POH and AHO are characterised by HO of intramembranous origin, predominantly at cutaneous and subcutaneous sites (Kaplan and Shore, 2000). While HO in the above conditions is driven by the underlying genetic disorder, it can also occur in response to triggering events, especially injury (Meyers et al., 2019). There are two such conditions collectively referred to as non-genetic or acquired disorders of HO, myositis ossificans traumatica (MOT) and neurogenic heterotopic ossification (NHO) (Meyers et al., 2019).

Amongst all these disorders, FOP is the most extensively studied. It is extremely rare, affecting 1 in 2,000,000 people (Baujat et al., 2017), has no ethnic predilection and is described as the most catastrophic among HO disorders in humans (Miao et al., 2012; Qi et al., 2017; Kaplan et al., 2008). Because it is so uncommon, FOP is frequently misdiagnosed during its initial stages and patients often experience a long gap between the onset of symptoms and ultimate diagnosis. According to the registry of the International FOP Association (IFOPA), the mean age at which the first symptoms occur is 5.4 years, while the mean age of FOP diagnosis is 7.5 years (Mantick et al., 2018). Although there has been remarkable progress in understanding the pathological mechanisms of FOP, it continues to present a significant clinical challenge. This article aims to outline the current information on FOP pathophysiology, clinical aspects, animal models, management strategies and future directions, and considers how knowledge of FOP can inform understanding of other more common forms of HO.

## Pathophysiology of FOP

### Gene mutations

**Fig. 1.**
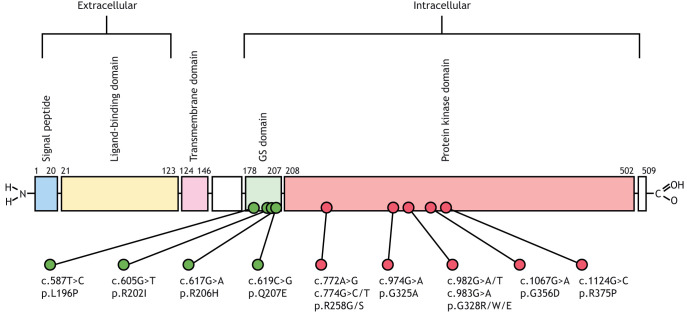
**Schematic representation of human ACVR1, its various domains and locations of the mutations that have been causally linked to FOP.** GS, glycine-serine.

All FOP patients reported to date were found to carry heterozygous gain-of-function mutations in the *ACVR1* gene, located on chromosome 2 (2q23-24). *ACVR1* encodes a bone morphogenetic protein (BMP) type 1 receptor, also known as activin A receptor type 1. In most cases, a single nucleotide transition (c.617G>A) causes a missense mutation of codon 206, resulting in substitution of arginine by histidine (R206H) in the intracellular glycine-serine (GS) domain of ACVR1 (Fig. 1). Atypical missense mutations (L196P, R202I, Q207E, R258G/S, G328R/W/E, G356D and R375P) in the GS or protein kinase (PK) domains of ACVR1 have also been identified in some FOP patients (Petrie et al., 2009; Katagiri et al., 2018a,b; Furuya et al., 2008; Huning and Gillessen-Kaesbach, 2014; Kaplan et al., 2009). In most cases, the mutations arise spontaneously *de novo*; however, a small number of FOP patients showing autosomal dominant inheritance from a symptomatic parent have also been reported (Kaplan et al., 1993; Connor et al., 1993; Shore et al., 2006).

### Dysregulated BMP signalling

**Fig. 2.**
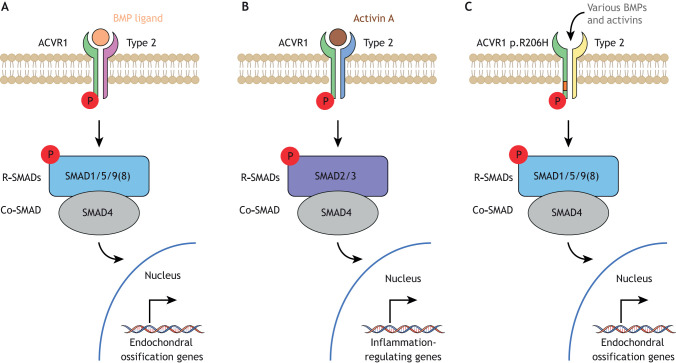
**BMP signalling.** (A) BMPs bind to complexes of type I and type II serine/threonine kinase BMP receptors, such as ACVR1, on the cell surface to activate intracellular signal transduction via R-SMADs SMAD1/5/9(8). Phosphorylated SMAD1/5/9(8) forms a complex with co-mediator SMAD4 and translocates into the nucleus, where it regulates transcription that drives endochondral ossification. (B) On binding activin A, complexes of type I and type II BMP receptors activate intracellular signal transduction via SMAD2/3, which activates a transcription programme that regulates inflammation. (C) ACVR1 carrying a FOP mutation (most frequently the R206H substitution) in the intracellular glycine-serine domain not only yields enhanced response to various BMP ligands by initiating downstream signalling via SMAD1/5/9(8), but also responds to various activin ligands, thereby favouring endochondral ossification by triggering an osteogenic gene expression programme. BMP, bone morphogenetic protein; Co-SMAD, common partner SMAD; P, phosphorylation; R-SMAD, receptor-regulated SMAD.

BMPs are required for multiple developmental processes (Wang et al., 2014), including bone and cartilage formation. Secreted BMPs bind to complexes of type I and type II serine/threonine kinase BMP receptors on the cell surface, such as ACVR1, to activate the intracellular signal transduction pathway (Fig. 2). In the absence of BMP ligands, the FK506-binding protein 1A (FKBP1A) binds to the GS domain of ACVR1 and inhibits the binding of effector molecules (Shen et al., 2009). Upon ligand binding, the type II receptor phosphorylates the type I receptor within its GS domain, releasing FKBP1A and thus allowing ACVR1 to bind and phosphorylate intracellular BMP-responsive transcription factors, the receptor-regulated SMADs (R-SMADs) SMAD1/5/9(8) (Wang et al., 2014). Phosphorylated SMAD1/5/9(8) forms a complex with the co-mediator SMAD4 that translocates into the nucleus, where it associates with co-activators or co-repressors to regulate transcription involved in endochondral ossification (Wang et al., 2014). Whereas SMAD1 and SMAD5 activate transcription in this context, SMAD9 acts as a transcriptional repressor (Tsukamoto et al., 2014). Notably, SMAD9 loss-of-function mutations lead to increased bone mineral density and cortical thickness, resulting in greater bone strength but not HO, as in FOP (Gregson et al., 2020).

Earlier studies of FOP suggested the activation of ACVR1^R206H^ in a ligand-independent manner, especially due to impaired binding of FKBP1A and thus inappropriate binding and phosphorylation of SMAD proteins, to be the predominant cause of HO (Shen et al., 2009). More recent work, however, established the role of inflammation in HO genesis and propagation in FOP patients (Alessi Wolken et al., 2018; Meyers et al., 2019). Alessi Wolken et al. suggested that the process by which HO occurs in FOP is likely to be ligand dependent and to involve ligands that activate ACVR1^R206H^, which are themselves regulated by inflammation (Alessi Wolken et al., 2018). Specifically, they showed that activin A, which is expressed by innate immune system cells and plays an important role in both promoting and resolving inflammation, is effectively perceived as a BMP ligand by ACVR1^R206H^, leading to downstream BMP signalling via SMAD1/5/9(8) (Alessi Wolken et al., 2018). Similarly, Hatsell et al. found that, as well as showing increased sensitivity to its ligands BMP2, BMP4, BMP7, BMP9 and BMP10, the mutant ACVR1^R206H^ also responded to activins A, AB, AC and B, to which the wild-type ACVR1 is unresponsive (Hatsell et al., 2015).

Activins, unlike BMPs, do not normally promote chondrogenesis or osteogenesis and generally signal via different SMADs, namely SMAD2/3 (Macias-Silva et al., 1998). In FOP, however, the mutated receptor erroneously perceives activin as a BMP, eliciting phosphorylation of SMAD1/5/9(8) as in normal BMP signalling (Sanchez-Duffhues et al., 2016). In addition to this mechanism, Wang et al. suggested that increased basal phosphorylated SMAD1/5/9(8) activity and local hypoxia occurring during tissue damage and inflammation can induce BMP signalling in a ligand-independent manner (Wang et al., 2018, 2016). Thus, tissue damage and inflammation in FOP patients can result in both ligand-dependent and -independent aberrant activation of the BMP signalling cascade, leading to activation of the endochondral ossification transcription programme by phosphorylated SMAD1/5/9(8).

### The cell of origin

The cells exhibiting dysregulated BMP signalling and osteogenic differentiation in FOP were originally thought to belong to the myogenic lineage (Katagiri et al., 2018a). However, the mesenchymal stem cell population at the site of inflammation has been found to be a more relevant source of progenitor cells that differentiate into chondrocytes and osteoblasts in response to aberrant signalling (Billings et al., 2008). The origin of these mesenchymal stem cells has been traced to local stromal/fibroblastic cells, endothelial cells (via endothelial-mesenchymal transition), Scx^+^ tendon progenitor cells, bone marrow-derived muscle-resident Mx1^+^ cells, glutamate transporter (Glast; also known as SLC1A3)-expressing progenitor cells and some circulating osteogenic precursor cells that can access bone-forming sites (Pignolo and Kassem, 2011; Ranganathan et al., 2015; Dey et al., 2016; Wosczyna et al., 2012; Pulik et al., 2020). This remains an area for further exploration and there are several ongoing studies in animal models to identify progenitor cell populations contributing to the development of HO (Lees-Shepard and Goldhamer, 2018).

### Metamorphosis

In FOP, soft tissue metamorphosis (Box 1) is the process by which skeletal muscle, tendons and ligaments transform into mature bone at various extraskeletal sites. There are two phases in this process: catabolic and anabolic. Inflammation represents the catabolic phase and is characterised by amplified infiltration of lymphocytes, macrophages and mast cells into the affected tissues, leading to necrosis (Kaplan et al., 2011). Since FOP patients have a pro-inflammatory baseline state characterised by increased pro-inflammatory and myeloid cytokines in their serum along with increased circulating pro-inflammatory monocytes, they naturally predispose to flare-ups (Box 1) (Barruet et al., 2018). In addition, experimental studies have shown that inflammatory triggers cause prolonged activation of the NF-κB signalling pathway in FOP monocytes and abnormal cytokine/chemokine secretion in both FOP monocytes and FOP primary monocyte-derived macrophages, likely mediated by NF-κB or p38 MAPK (also known as MAPK14) activity (Barruet et al., 2018). These factors seem sufficient to drive the catabolic phase in FOP. Following this, an anabolic phase supervenes, characterised by fibroproliferation, neovascularity and angiogenesis (Pignolo et al., 2005). The pool of mesenchymal cells at the site of inflammation undergoes differentiation in response to the activation of the BMP signalling cascade, leading to transformation of the fibroproliferative tissue into cartilage, which in turn matures into bone through an endochondral process, thus completing the process of metamorphosis (Kaplan et al., 2011).

## Diagnosis and management

Identification of the congenital and episodic signs and correlating them to FOP is of utmost importance (Box 2). This is sufficient for a working diagnosis of FOP. However, a high index of suspicion is needed to make a diagnosis based solely on clinical presentation. A lack of suspicion leads to delayed diagnosis or misdiagnosis, which potentiates inappropriate and unnecessary testing, especially invasive biopsies that may cause flare-ups and actually promote HO. Hence, clinicians, especially paediatricians, who are typically the first to come across children with FOP, need to be aware of this condition, its consequences and effective mitigating strategies. Even though biochemical and radiological investigations can provide useful information on the disease process, any kind of invasive procedure is contraindicated as it induces local inflammation and eventually HO; hence, biopsy of lesions should never be attempted (Trigui et al., 2011). Ultimately, the diagnosis can only be confirmed by DNA sequence analysis to trace the underlying mutation.

**Box 2. Clinical case presentation – a consolidated summary from the literature**

**Classical signs**

Most patients with FOP are typically born with congenital great toe malformations such as hallux valgus and macrodactyly (Box 1). Signs of HO start to occur episodically from the first decade of life. Usually, painful and warm soft tissue swellings are the first to appear, a stage referred to as a ‘flare-up’ (Alessi Wolken et al., 2018). Flare-ups are generally sporadic and unpredictable, and are caused by the underlying inflammation in the ligaments, tendons or skeletal muscle occurring upon pro-inflammatory insults such as muscle fatigue, tissue damage, intramuscular injections or viral illness. Over time, owing to the repeated flare-ups at different sites, progressive and cumulative ossification of soft tissues occurs, leading to the debilitating effects of FOP (Kaplan et al., 2012; Pignolo et al., 2013).

**Atypical features**

In addition to the two classical signs (hallux valgus and macrodactyly), some FOP patients present with one or more atypical signs and are categorised as FOP-plus. These atypical signs include tibial osteochondromas (Box 1), spinal malformations and broad femoral neck, which are usually reported among patients with R206H and Q207E missense mutations, and thumb malformations, cognitive impairment and diffuse scalp thinning, which are reported among patients with other atypical missense mutations in ACVR1 (Qi et al., 2017). Several recent reports have also documented delayed-onset HO and absence of characteristic great toe malformations among patients with atypical mutations, who are thus categorised as FOP variants (Jiao et al., 2013).

**Debilitating effects**

Starting with neck stiffness and a local flare-up episode, the cervical spine becomes involved early during the course of the disease (Pignolo et al., 2011). At this stage, radiographs of the cervical spine might reveal large posterior elements, tall and narrow vertebral bodies and fused facet joints. Eventually, neck movements become completely restricted due to bridging bone formation across segments, referred to as bony ankylosis (Box 1) (Katagiri et al., 2018b). Similar episodes occur in no particular order throughout the body, resulting in ankylosis of various joints, and most patients become wheelchair bound by the end of the second decade of life (Kaplan et al., 2010). Some patients may experience hearing loss due to middle ear ossification (Levy et al., 1999). The involvement of the jaw can lead to feeding difficulties, resulting in malnourishment and gradual weight loss, which can be addressed with feeding assistance (Pignolo et al., 2011). However, the debilitating effects of FOP start to become life threatening with the involvement of intercostal muscles, costovertebral joints and thoracic paravertebral soft tissues, which, when ossified, result in thoracic insufficiency syndrome (Box 1) (Kaplan and Glaser, 2005). Patients eventually die due to resulting complications such as pneumonia or right-sided heart failure. FOP patients have a median life span of around 40 years (Kaplan et al., 2010).

As the entire cascade of events leading to HO is triggered by tissue damage and subsequent inflammation, prevention and control of inflammation are the basis of clinical management. However, preventing a triggering event is extremely challenging, as such events could be anything from significant trauma to trivial ocurrences such as intramuscular injections, blunt muscle trauma from bumps, bruises and falls, influenza-like illnesses and, in some cases, mere muscle fatigue (Kaplan et al., 2008). How each of these events influence HO is not fully understood, mainly because they remain unnoticed until a flare-up occurs. However, since inflammation follows every trigger, clinical management is mainly focused on mitigating inflammation, thereby alleviating symptoms. As yet, there is no definitive treatment that can alter the natural course of FOP.

According to Kaplan et al. (2019) the pharmacological agents for managing FOP based on research findings and anecdotal experience can be divided into three classes (Table 1). Class I medications are those that contain acute inflammation flare-ups. These include high-dose corticosteroids and non-steroidal anti-inflammatory drugs (Kaplan et al., 2019; Pignolo et al., 2013; Qi et al., 2017). Class II medications are used for management of other conditions but have a theoretical application in FOP (Kaplan et al., 2019; Schaper et al., 2011; Convente et al., 2018; Werner et al., 2013; Brantus and Meunier, 1998; Pennanen et al., 1995; Pabst et al., 2014; Brennan et al., 2018). These are being used anecdotally as adjunctive therapy for refractory flare-ups in FOP (Kaplan et al., 2019). However, there is as yet no solid evidence to substantiate the use of these drugs. Finally, class III medications are those that are currently under clinical investigation as holding the key for an effective treatment (Kaplan et al., 2019; IFOPA, 2020). These drugs are mainly focused on inhibiting the canonical and non-canonical BMP signalling from ACVR1^R206H^ at both the extracellular and intracellular levels. Other proposed pre-clinical approaches to inhibit mutant ACVR1-dependent bone induction include the use of nucleic acid-based inhibitors, such as allele-specific RNA interference or exon-skipping oligonucleotides, BMP receptor kinase inhibitors (such as dorsomorphin), downstream BMP signalling inhibitors (for example, Fendiline and Perhexiline) and fungal metabolite osteoblast differentiation inhibitors (such as NG-391, NG-393 and Trichocyalide A/B) (Katagiri et al., 2018a; Yamamoto et al., 2013; Sanvitale et al., 2013; Fukuda et al., 2012; Cappato et al., 2018).

**Table 1.**
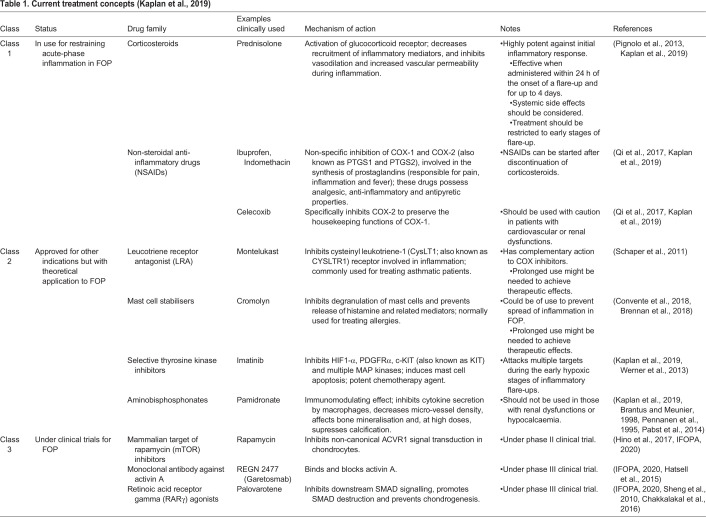
**Current treatment concepts (Kaplan et al., 2019)**

## Animal models in FOP research

One of the first animal models to provide insights into the role of BMP signalling in HO was established prior to the discovery that FOP patients carry activating mutations in ACVR1. Transgenic mice overexpressing BMP4 under the control of neuron-specific enolase were initially developed to study the role of BMP signalling in brain development (Gomes et al., 2003). However, by 2 months of age, these mice started showing phenotypes such as stiffness and gait abnormalities (Kan et al., 2004). Histological and immunohistochemical analysis of limb tissue showed muscle degradation and proliferation of subcutaneous fibroblast-like cells (Kan et al., 2004). Subsequently, HO of endochondral origin was found at multiple locations, such as hind limbs, fore limbs, abdominal wall and paravertebral regions, with concomitant skeletal deformities and restriction of movements (Kan et al., 2004; Kan and Kessler, 2011). Other approaches to inducing ectopic BMP activity, such as knocking out BMP inhibitors or overexpressing BMP target genes, however, failed to phenocopy FOP, and subsequent models have focused on the expression of mutant ACVR1 (Kan and Kessler, 2011).

Functional orthologues of human ACVR1 are found throughout the animal kingdom, and expression of the classical or atypical FOP mutant forms of the receptor has been shown to cause dysregulated BMP signalling in several laboratory animals, including *Drosophila*, mouse, chick and zebrafish (Twombly et al., 2009; Chakkalakal et al., 2012; Haupt et al., 2014; LaBonty and Yelick, 2018) (Table 2). Among these commonly used animals, the mouse ACVR1 is 98.4% identical to its human orthologue, whereas the zebrafish ACVR1 (Acvr1l) shows only 69% identity. However, the GS and kinase domains of the zebrafish receptor are more similar (85% identity) (Yelick et al., 1998; LaBonty and Yelick, 2018), validating the use of both these model organisms for studying FOP. Because normal ACVR1 activity is required for gastrulation, neural crest differentiation and germ cell development in mouse, and for dorsal-ventral patterning in zebrafish, embryonic expression of the FOP-associated mutation results in lethality (Chakkalakal et al., 2012; LaBonty et al., 2017). To circumvent this problem, conditionally expressed mutant forms of ACVR1 have been developed to model FOP in both mouse and zebrafish (Table 2) (Yu et al., 2008; Chakkalakal et al., 2012; Hatsell et al., 2015; LaBonty et al., 2017; LaBonty and Yelick, 2018).

**Table 2.**
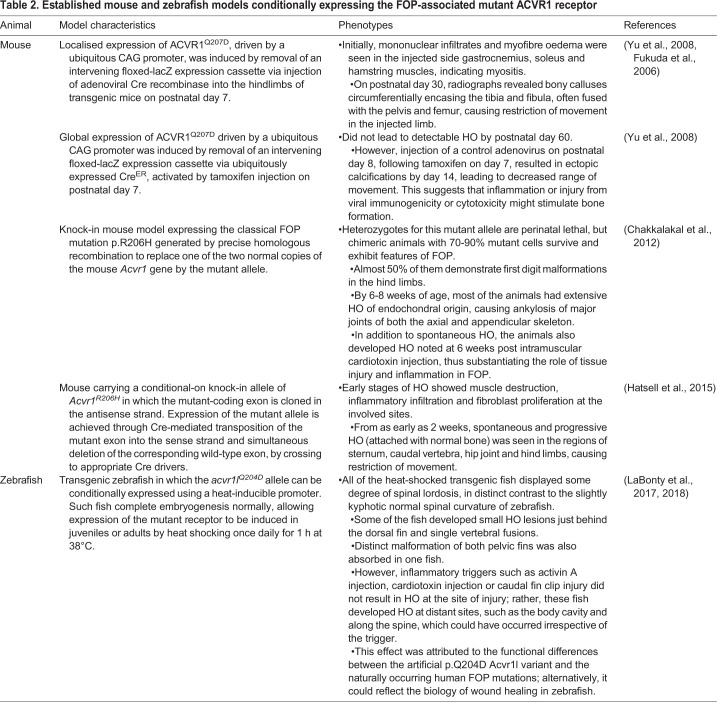
**Established mouse and zebrafish models conditionally expressing the FOP-associated mutant ACVR1 receptor**

These transgenic animal models have paved the way not only to identifying the cell of origin and the pathomechanisms of FOP, but also to implementing further pre-clinical testing of novel medical interventions. The discovery that retinoic acid receptor (RAR) agonists can prevent the stimulatory effect of RARs on SMAD-mediated transcription (Lees-Shepard et al., 2018; Chakkalakal et al., 2016; Shimono et al., 2011; Sheng et al., 2010) prompted testing of palovarotene, a RARγ agonist, in animal models. Its efficacy in repressing chondrogenesis, cartilage formation and subsequent HO was initially demonstrated *in vivo* in two of the FOP models described above, the Cre-inducible constitutively active ACVR1^Q207D^ mouse model and the genetically humanised conditional-on knock-in mouse harbouring the classical ACVR1^R206H^ mutation (Fukuda et al., 2006; Chakkalakal et al., 2016). Work in juvenile FOP mice also showed that daily dosing with palovarotene prior to skeletal maturity could result in long bone growth plate ablation, suggesting that the developmental stage, duration of exposure and dosing interval need to be optimised for safe and effective use of palovarotene without complications (Lees-Shepard et al., 2018). Another concept of inhibiting activin A with a blocking antibody, the basis of the clinical candidate REGN2477 (garetosmab), was first demonstrated in the genetically humanised conditional-on knock-in mouse model of FOP that showed neither spontaneous nor injury-mediated HO (Hatsell et al., 2015). Furthermore, Rapamycin, which is currently the subject of a clinical trial, was tested in ACVR1^R206H^ mice and in a FOP-induced pluripotent stem cell-based HO model in which ectopic bones derived from FOP patient-derived cells are formed in mice. In both models, treatment with Rapamycin reduced HO (Agarwal et al., 2016; Hino et al., 2017). Overall, animal studies have contributed significantly to the understanding and management of FOP. Moreover, the need for accurate animal models remains, as more therapeutic modalities that target and regulate multiple mechanisms of the BMP signalling cascade in FOP are constantly being designed.

## Future directions

FOP research is progressing towards translational success. Animal models have helped unravel its pathobiology: it is now evident that inflammation, dysregulation of BMP signalling and endochondral ossification are key processes contributing to HO in FOP patients. One focus of current research is on discovering more ways to redirect the progenitor cells in the inflammatory environment away from adopting an osteogenic fate towards more of a soft tissue fate. However, there is also a debate as to whether aberrant BMP signalling is solely responsible for HO in FOP (Kan et al., 2018). Given the role of Hedgehog (Hh) signalling, mediated predominantly via Indian Hh in normal osteogenesis, especially the differentiation of chondrocytes during endochondral ossification (Martelli and Santos, 2014; Lai and Mitchell, 2005; St-Jacques et al., 1999; Long et al., 2004), it is plausible that this pathway may also contribute to HO in FOP, which has yet to be explored in detail. Similarly, the Wnt/β-catenin signalling pathway, which is thought to influence the differentiation and function of mesenchymal stem cells, chondrocytes, osteoblasts and osteoclasts during normal bone formation, may also have a role (Regard et al., 2012). Although mutations in these pathways have not been found in patients with FOP, it is possible that they could influence HO through crosstalk with aberrant BMP signalling seen in FOP. Indeed, a recent study demonstrated that genetic removal of Hh can abolish HO in mouse models, not only POH, but also FOP and acquired HO. The authors identified ‘Hedgehog-driven, self-amplifying osteoblast differentiation as a common cellular and molecular mechanism underlying HO initiation and expansion’, suggesting a new therapeutic focus (Yang, 2020). The possible involvement of Hh and Wnt signalling pathways in FOP and other forms of HO certainly warrants further investigation.

## Conclusion

The aetiology of FOP has long been an unsolved puzzle; however, years of extensive research are bringing us closer to a full understanding of this distressing and debilitating condition. Exploration of FOP has informed our understanding of the BMP signalling cascade, cellular response to inflammation and connective tissue metamorphosis, leading to the development of promising new therapeutic strategies that are the subject of ongoing clinical trials. Since lesions in acquired HO disorders such as MOT and NHO often present a combination of endochondral ossification, as in FOP, and intramembranous ossification, as in POH, progress in understanding and treating these rare diseases could hold the key for developing management strategies and effective treatment for the far more prevalent, yet clinically puzzling, acquired forms of HO. Progress in understanding FOP has been made possible through the growing FOP research network that has overcome the fundamental challenges of rare diseases: creating awareness, maintaining patient registries, providing education and support for patients and families, conducting clinical trials and sharing information. In this way, FOP research provides a model for other rare disease communities to overcome these challenges through active collaboration between patients, researchers and advocates.
